# Development and validation of a classification algorithm to diagnose and differentiate spontaneous episodic vertigo syndromes: results from the DizzyReg patient registry

**DOI:** 10.1007/s00415-020-10061-9

**Published:** 2020-07-13

**Authors:** Michael Groezinger, Doreen Huppert, Ralf Strobl, Eva Grill

**Affiliations:** 1 Institute for Medical Information Processing, Biometry and Epidemiology, Ludwig-Maximilians-Universität München (LMU), Marchioninistr. 15, 81377 München, Germany; 2 German Centre for Vertigo and Balance Disorders, University Hospital Munich, Campus Grosshadern, Munich, Germany; 3 Department of Neurology, Ludwig-Maximilians-University, Munich, Germany

**Keywords:** Vestibular migraine, Menière’s disease, Vestibular disease, Machine learning, Classification

## Abstract

**Background:**

Spontaneous episodic vertigo syndromes, namely vestibular migraine (VM) and Menière’s disease (MD), are difficult to differentiate, even for an experienced clinician. In the presence of complex diagnostic information, automated systems can support human decision making. Recent developments in machine learning might facilitate bedside diagnosis of VM and MD.

**Methods:**

Data of this study originate from the prospective patient registry of the German Centre for Vertigo and Balance Disorders, a specialized tertiary treatment center at the University Hospital Munich. The classification task was to differentiate cases of VM, MD from other vestibular disease entities. Deep Neural Networks (DNN) and Boosted Decision Trees (BDT) were used for classification.

**Results:**

A total of 1357 patients were included (mean age 52.9, SD 15.9, 54.7% female), 9.9% with MD and 15.6% with VM. DNN models yielded an accuracy of 98.4 ± 0.5%, a precision of 96.3 ± 3.9%, and a sensitivity of 85.4 ± 3.9% for VM, and an accuracy of 98.0 ± 1.0%, a precision of 90.4 ± 6.2% and a sensitivity of 89.9 ± 4.6% for MD. BDT yielded an accuracy of 84.5 ± 0.5%, precision of 51.8 ± 6.1%, sensitivity of 16.9 ± 1.7% for VM, and an accuracy of 93.3 ± 0.7%, precision 76.0 ± 6.7%, sensitivity 41.7 ± 2.9% for MD.

**Conclusion:**

The correct diagnosis of spontaneous episodic vestibular syndromes is challenging in clinical practice. Modern machine learning methods might be the basis for developing systems that assist practitioners and clinicians in their daily treatment decisions.

**Electronic supplementary material:**

The online version of this article (10.1007/s00415-020-10061-9) contains supplementary material, which is available to authorized users.

## Introduction

Spontaneous episodic vertigo syndromes—vestibular migraine (VM) as the most frequent form of episodic vertigo with a lifetime prevalence of 1% [1] and Menière’s disease (MD) with a prevalence of about 0.2–0.5%—are difficult to differentiate, even for an experienced clinician after a thorough anamnesis and clinical examination. Both VM and MD can have considerable impact on patients’ daily life and functioning, so timely and appropriate therapy is essential. The diagnostic criteria for VM and MD correspond to core symptoms of these diseases from the literature for VM [2–10], and for MD [11]. These diagnostic criteria were formulated by the International Bárány Society for Neuro-Otology in 2012 [12] and 2015 [13] (Table 1) Epidemiological studies revealed a coincidence of both conditions in one individual [14–16] which may pose considerable uncertainties in the evaluation of the response of medical treatment.

**Table 1:** Current definitions of Vestibular migraine and Menière’s disease

Vestibular migraine	At least 5 episodes with vestibular symptoms of moderate or severe intensity, lasting 5 min. to 72 h Current or previous history of migraine with or without aura according to the International Classification of Headache Disorders (ICHD) One or more migraine features with at least 50% of the vestibular episodes: headache with at least two of the following characteristics: one-sided location, pulsating quality, moderate or severe pain intensity, aggravation by routine physical activity photophobia and phonophobia visual aura Not better accounted for by another vestibular or ICHD diagnosis Probable: A, B or C, and D
Menière’s disease	Two or more spontaneous episodes of vertigo, each lasting 20 min to 12 h Audiometrically documented low- to medium-frequency sensorineural hearing loss in one ear, defining the affected ear on at least one occasion before, during or after one of the episodes of vertigo Fluctuating aural symptoms (hearing, tinnitus, or fullness) in the affected ear Not better accounted for by another vestibular diagnosis Probable: A, C, and D

Adapted from Lempert et al. 2012 [12] for vestibular migraine and Lopez-Escamez et al. 2015 [13] for Menière’s disease

Arguably, clinicians with longstanding expertise and experience will be able to integrate all these diagnostic dimensions. In situations of routine primary care, however, where this expertise is not easily available, diagnostic information will often be too complex to be summarized into one clinical decision. In the presence of seemingly overwhelming information, automated systems can support human decision making. This will be quite straightforward if the logic of the decision-making process is known. However, in the absence of an existing algorithm, diagnosis based on clinical expert experience is a process that is hard to describe.[17].

Several attempts have been made to facilitate bedside diagnosis of vestibular diseases using machine learning (ML) approaches. Some of these approaches were able to classify specific diseases with good accuracy. Interestingly, in these ML studies MD was persistently difficult to predict [18, 19]. As a further development, a combination of several data mining techniques has recently been proposed for 12 vestibular diseases, MD among these, however, without specific solutions for single hard-to-differentiate disease entities [20]. Another study was able to differentiate unilateral canal damage, one potential feature in MD, with 76% accuracy [21]. Combining these promising approaches using recent developments in ML, might be even more successful [22].

Objectives of this study were to develop and test a classification algorithm to differentiate Vestibular Migraine and Menière’s disease in clinical practice.

## Methods

Our data originates from the DizzyReg patient registry of the German Center for Vertigo and Balance Disorders (DSGZ). Details on purpose and data collection have been published elsewhere [23, 24]. In brief, the registry is a prospective data base including all relevant anamnestic, sociodemographic, diagnostic and therapeutic information of patients that presented at the DSGZ, a specialized tertiary treatment center at the hospital of the LMU Munich with approximately 3000 patients per year since 2016.

A positive vote of the local institutional review board and detailed consulting on data protection issues from the regional data protection officer was obtained for the registry. Informed consent was obtained from each patient or the patient’s legal surrogate.

Patients received a thorough neuro-otologic assessment and a validated standardized diagnosis according to current international guidelines [12, 25]. Clinicians with longstanding experience diagnosed all patients. This clinical diagnosis represents the gold standard for our study.

DizzyReg centralizes all data that is collected in electronical health records or medical discharge letters. The data is stored on servers within the hospital firewall and state-of-the-art security techniques are used to protect the data. The data is either retrieved online from the clinical workplace system (CWS) or entered manually via a web-based system. It will only be released fully anonymized only for predefined purposes after review of an external steering group [23].

Reporting of methods follows the guidelines by Luo et. al.[26].

The classification task was to differentiate cases of VM, MD from other vestibular disease entities. As the number of VM and MD cases was relatively small, we decided to formulate two separate one-vs-all models, one for the prediction of VM and one for the prediction of MD. This approach has been used successfully before [19].

We defined precision (positive predictive value) and sensitivity as criteria to judge the quality of the classification. Sensitivity is the probability of the ML classifier to classify a patient as having MD or VM when this disease is truly present, i.e., the number of correctly classified cases (the true positives TP) divided by the total number of persons with this disease. Precision is the number of correctly classified cases among all cases diagnosed by the algorithm. Additionally, the F-measure [27] was calculated as a combination of precision and sensitivity and as a combination of several thresholds of sensitivity and specificity, respectively.

$$ {\text{Precision } = \text{ }}\frac{{{\text{ true positives }}\left( {{\text{TP}}} \right)}}{{{\text{TP } + \text{ false positives }}\left( {{\text{FP}}} \right)}} $$

$$ {\text{Sensitivity } = \text{ }}\frac{{\text{ TP}}}{{{\text{TP } + \text{ false negatives }}\left( {{\text{FN}}} \right)}} $$

$$ {\text{Specificity } = \text{ }}\frac{{{\text{ true negatives }}\left( {{\text{TN}}} \right)}}{{\text{TN } + \text{ FP}}} $$

$$ {\text{Accuracy } = \text{ }}\frac{{\text{ TP } + \text{ TN}}}{{\text{TP + TN + FP + FN}}} $$

$$ F{\text{ - measure = }}\frac{{{ 2*}\left( {\text{precision } + \text{ sensitivity}} \right)}}{{\text{precision*sensitivity}}} $$

To build the classification model we first identified candidate predictors that were statistically associated with presence or absence of either VM or MD. Selection was based on VM vs. MD. The initial data set contained 582 variables. Examples of these variables along with their clinical categories is available in the electronic supplement. Variables would be candidates if they had a p-value of 0.2 or below in bivariate chi-square tests (for categorical variables) or Mann–Whitney-Tests (for metric variables). This analysis revealed 105 variables that were then used as input for the subsequent models. Table 1 of the supplementary material lists a summary of all variables used for training.

Variables were then examined for missingness and those with insufficient information (missingness above 90%) were not included in the analyses. Likewise, variables with little variation, and redundant variables were deleted. This reduced the number of variables to 96.

A total of 20 variables had missing values. Overall, 8446 values were missing (6.2%), all patient records had at least one missing value. These remaining 20 variables were imputed.

In line with current recommendations we used multiple imputation techniques for missing data [28], namely Multivariate Imputation by Chained Equations (MICE) [28, 29]. In brief, MICE specify a multivariate imputation model on a variable-by-variable basis by a set of conditional distributions. From those conditional distributions imputed values are drawn with Markov Chain Monte Carlo techniques. To acknowledge uncertainty in the imputation process MICE yields five different imputed data sets that were then used for training the classification models [28].

Deep Neural Networks (DNN) were used for classification. In brief, neural networks are computational graphs which perform compositions of simpler functions to provide a more complex function used for separation in classification tasks. This type of model has proven to very successful in classification tasks in medical imaging.

DNN provide a wide range of meta parameters which then need to be tuned to optimize the classification results. To limit the computing effort, we relied on recommended standards for meta-parameters and varied breadth (number of nodes per hidden layer) and depth (number of hidden layers) to examine their impact on classification behavior. For a shallow network we trained one hidden layer with 10 different numbers of nodes, each 10 times. The best configuration was selected based on the F-measure. To test the impact of a second hidden layer, we added another hidden layer (10 different number of nodes and 10 training runs). The best configuration for the two layers was again selected by F-measure. For the deep configuration we started with 4 hidden layers and added four additional ones and changed the number of nodes similarly to the shallow configuration.

To identify the best model configuration in terms of breadth vs. depth of the DNN we first used a shallow configuration of the network (a maximum of two hidden layers). After optimizing breadth for the first layer, a second hidden layer was added and optimized. This yielded the best shallow configuration. Then we extended the depth of the network further into a deep configuration with a maximum of eight hidden layers. Deep networks generally do not need the same breadth as shallow configurations to achieve adequate training results, we still kept the breadth in the deep configuration to 1000 nodes per hidden layer. To obtain unbiased estimation of the quality criteria an independent validation set was defined by setting aside 20% of the participants. This validation set was used to estimate accuracy, precision, specificity and F-measure. This was repeated ten times with a different randomly drawn validation set.

As sample size was relatively small and we wanted to improve training results we then repeatedly trained the whole network again, similar to the work by Erhan [30–33]. We conducted an initial training run, kept the obtained weight parameters, and trained the network again but with a new shuffled random train/test data split. This was used for the best shallow and the best deep network configuration from the initial DNN. The pre-training workflow is described in Fig. 1 of the supplementary material.

**Fig. 1:**
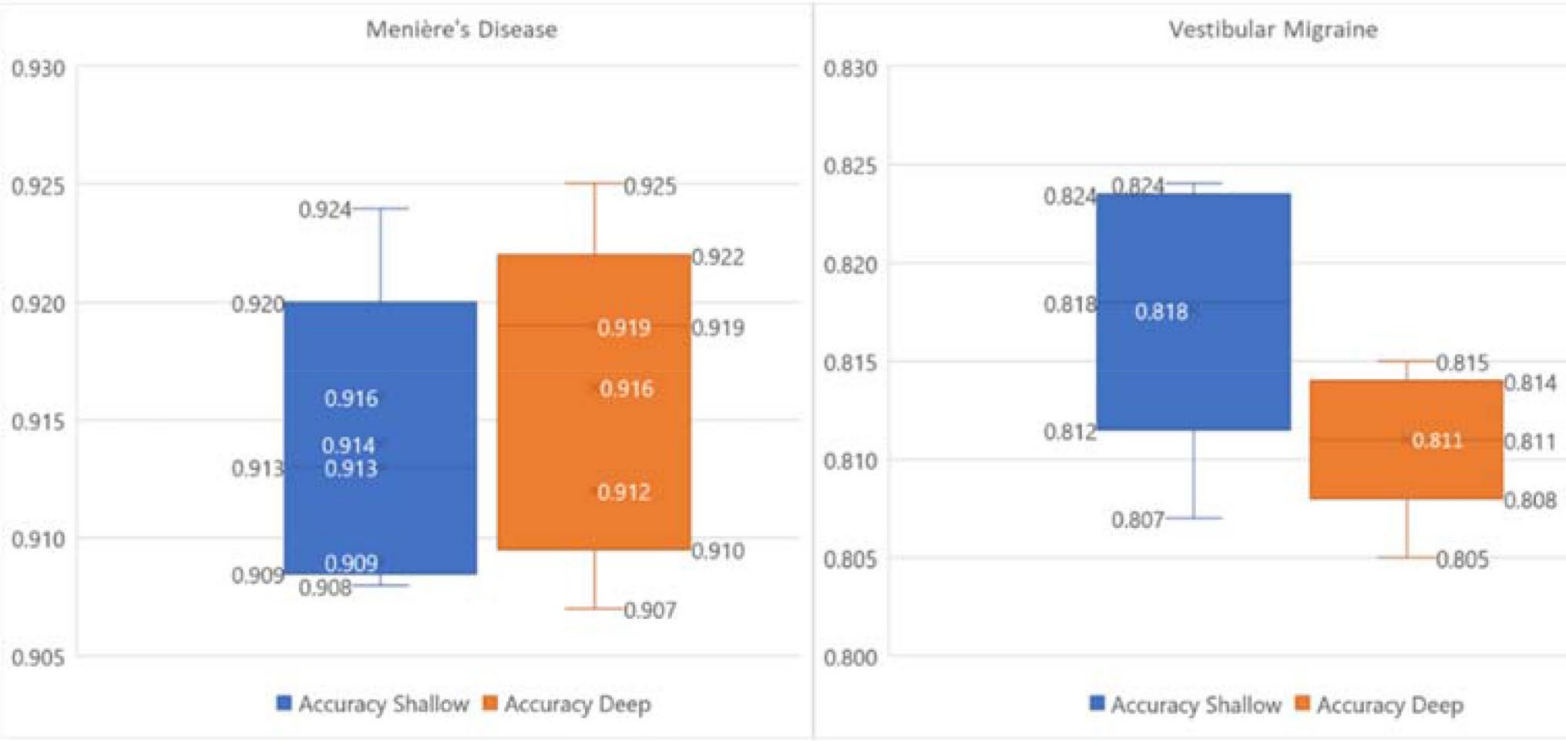
Distribution of the test accuracy predicting Menière’s disease and vestibular migraine over 5 imputed data sets for shallow and deep configurations of the Deep Neural Network model

Additionally, boosted decision tree (BDT) models were applied [34]. In contrast to DNN, these models yield information on the relative importance of single variables for the model [35]. To identify the best model configuration, we applied sensitivity analyses for the maximum number of trees in the model while keeping all other model parameters fixed (see Appendix II). Training and evaluation was carried out for all five imputed data sets and for both VM and MD as binary outcomes, i.e., prediction of VM or MD vs. all other diagnoses.

We implemented the models in the Python programming language, supported by the TensorFlow library v1.15.

## Results

A total of 1357 patients were included (mean age 52.9, SD 15.9, 54.7% female), among these 9.9% with MD and 15.6% with VM. The most frequent other diagnoses were benign paroxysmal positional vertigo (10.4%), chronic unilateral vestibular failure (7.0%), bilateral vestibular failure (5.1%), and polyneuropathy (4.5%).

Shallow Deep Neural Networks (DNN) classified MD with a mean F-measure 55.5 ± 2.7% across all five imputed data sets (accuracy 91.4 ± 0.6%, precision 55.4 ± 0.6%, sensitivity 54.7 ± 2.8%). Using pre-training on the best shallow network configuration improved F-measure to 90.0 ± 4.6%, accuracy to 98.0 ± 1.0%, precision to 90.4 ± 6.2% and sensitivity to 89.9 ± 4.6%. Figure 1 shows results averaged over all imputed data sets. Figure 3 depicts the results for pre-training for MD and their values over the number of training runs.

**Fig. 2:**
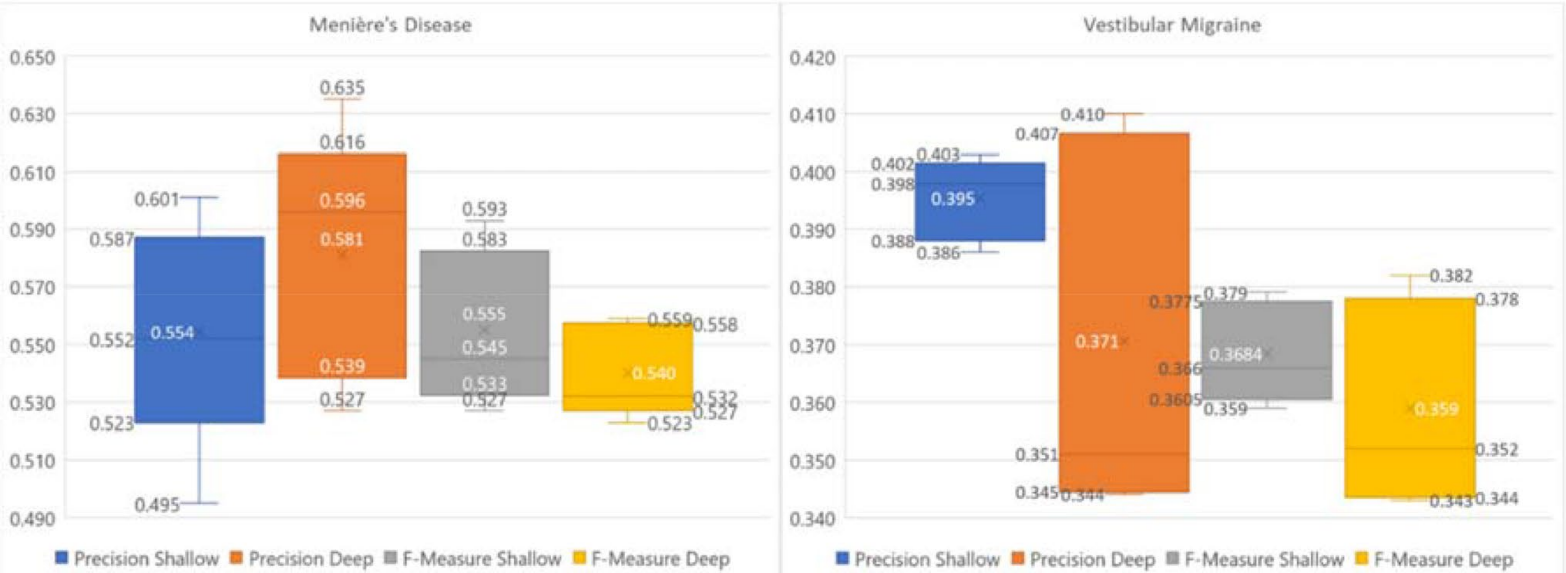
Distribution of the test accuracy predicting Menière’s disease and vestibular migraine over 5 imputed data sets for shallow and deep configurations of the Deep Neural Network model

**Fig. 3:**
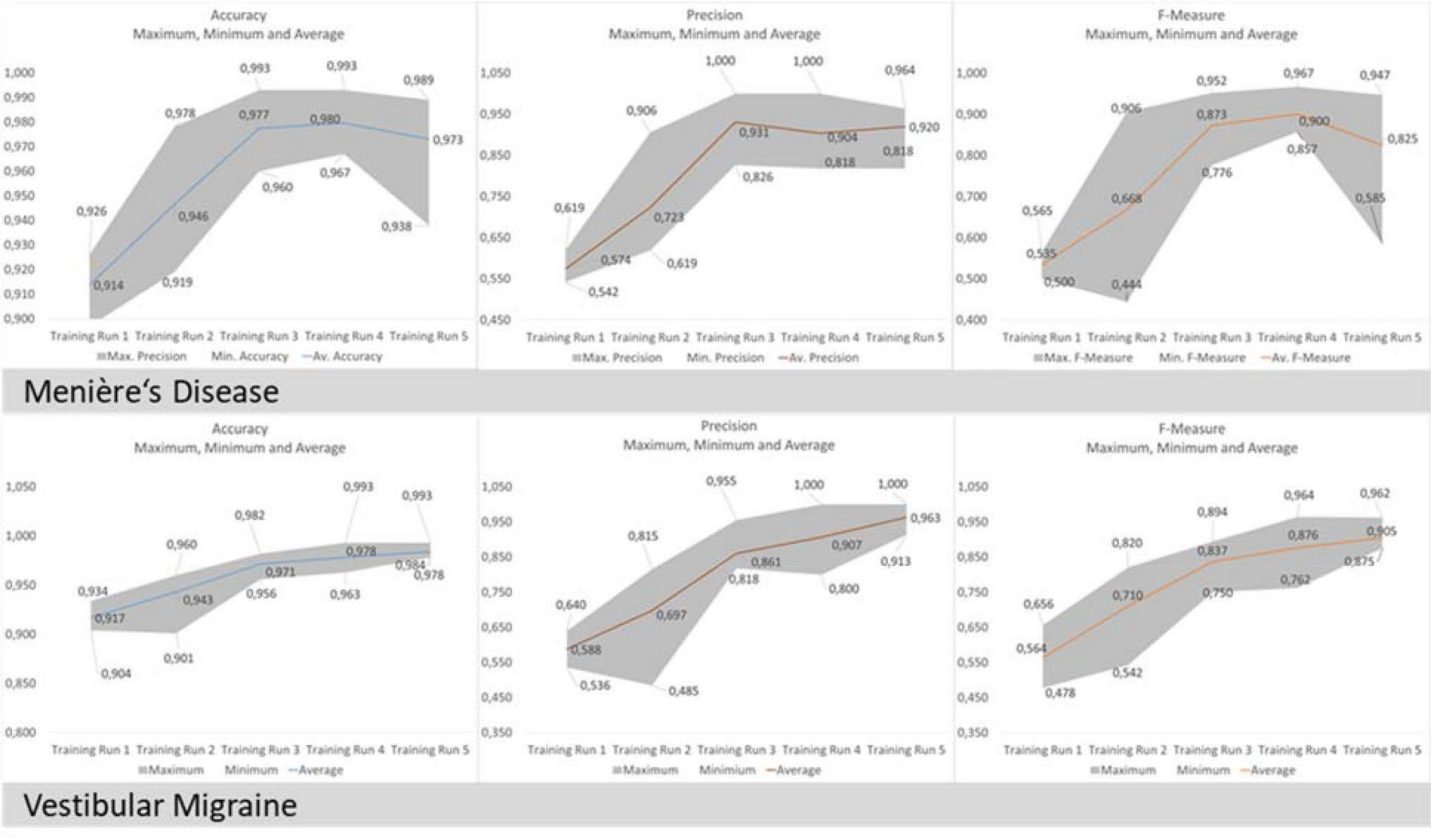
Accuracy, precision and F-measure after pre-training for Menière’s disease with a shallow network configuration (layer 1/2: 200/50 nodes) and for vestibular migraine with a deep network configuration (4 layers/50 nodes each). The lines represent means, the borders of grey areas represent minimum and maximum values over all five imputed data sets

The shallow configuration classified VM yielding a mean F-measure of 36.8 ± 0.8% (accuracy 81.8 ± 0.6%, precision 39.5 ± 0.6%, sensitivity 35.1 ± 2.7%). Pre-training using the best deep configuration (4 layers 50 nodes) improved classification considerably yielding a F-measure of 90.5 ± 3.0% (accuracy 98.4 ± 0.5%, precision 96.3 ± 3.9%, sensitivity 85.4 ± 3.9%,). Results of shallow and deep configurations as means of the five imputed data sets are shown in Fig. 2. Pre-training results per run VM are shown in Fig. 3.

Boosted decision trees for Menière’s disease yielded a mean F-measure 53.3 ± 3.0%, (accuracy of 93.3 ± 0.7%, precision 76.0 ± 6.7%, sensitivity 41.7 ± 2.9%). Vestibular migraine was classified with a mean F-measure 27.6 ± 5.1% (accuracy of 84.5 ± 0.5%, precision 51.8 ± 6.1%, sensitivity 16.9 ± 1.7%) (Table 2). After training, BDT provide a listing of features according their importance in the decision process. Table 2 lists the 10 most important variables for Menière’s disease and vestibular migraine, respectively.

**Table 2:** Results of boosted decision tree models. We show the variables with the highest importance for prediction of Menière's disease and vestibular migraine identified by a boosted decision tree

Menière’s disease		Vestibular migraine		
Variable	Importance	Variable		Importance
Age	9,13%	Age		11,94%
Caloric side difference	7,60%	Caloric side difference		7,06%
Patient reported vomiting	7,12%	Gain left (ms)		6,62%
Vertigo lasting several hours	4,92%	Gain right (ms)		5,68%
Gain left (ms)	4,63%	Anamnestic headache		2,62%
Patient reported hearing loss	4,38%	Hours of sleep in 24 h		1,91%
Impaired hearing (audiometry)	2,90%	Visual acuity		1,61%
Hours of sleep in 24 h	2,84%	Patient reported nausea		1,55%
Gain right (ms)	2,56%			
Visual acuity	2,33%			

## Discussion

Differentiating Menière’s disease (MD) from vestibular migraine (VM) is a challenge when patients present with episodic vertigo. The Deep Neural Network architecture of this study, augmented by pre-training, showed excellent classification performance for both MD and VM. This result was achieved with a relatively small set of variables, making the system potentially available for applications that run in non-expert settings.

The accuracy of the networks reported here exceeds that of previous models at large. A study using Support Vector Machines and k-Nearest Neighbour reported an overall accuracy of 79.8% [19] for a model differentiating MD from 8 other vestibular syndromes, but this model did not include patients with VM. Likewise, a Support Vector Machines approach to classify unilateral vestibulopathy yielded an accuracy of 76% [21]

The results indicate that DNN perform better to classify MM than to classify VM. Indeed, the differentiation of VM from other episodic vertigo syndromes may be difficult, especially when it comes to differentiating MD and VM. It is important to stress that up to 60% of patients with MD also fulfill some or all of the diagnostic criteria for VM and vice versa [15]. A combination of ear symptoms like a ringing in the ears and dizziness is among the current diagnostic criteria for MD [13]. Another factor that complicates the differentiation of VM and MD is the absence of headache. Interestingly, a history of headache was among the more prominent features that indicated VM in our study. Differentiation will be even more difficult if attacks of MD occur without ear symptoms, which is especially frequent at the beginning of MD [11]. Moreover, both diagnoses (uni- or bilateral Menière’s disease and migraine with and without aura) coincided in 56% compared with 25% in an age-matched control group [14–16].

One major disadvantage of DNN is the “black box” approach, because DNN do not yield any information about the relative importance of single variables to the model. Thus, DNN are not particularly useful to create parsimonious models. We, therefore, applied a different machine learning method, boosted decision trees, firstly to compare their performance to that of DNN, but secondly to investigate variable importance that can be useful to concentrate on characteristics with high value for clinical decision-making.

For both MD and VM, caloric side difference and gain of the head impulse test were important predictors derived from boosted decision trees. Most patients with VM have mild central ocular motor disorders in the form of gaze-evoked nystagmus, saccadic smooth pursuit eye movements, or a positional nystagmus even in the attack-free interval [5, 36–39]. Regarding predictors that do not rely on instrumental tests, patient reported hearing loss, vomiting and a duration of attacks of several hours were the most striking characteristics for MD, while a history of headache and patient reported nausea were indicative for VM. These results are not completely surprising, given the typical features of hearing loss for MD and headache for VM. Nevertheless, they can be used for further refinement of diagnostic algorithms.

We acknowledge several limitations. First, identification of MD and VM relied on the clinical diagnoses that were made in a tertiary care center. While these diagnoses are certainly accurate, there might be complex cases, where a definite diagnostic decision can only be made after follow-up. On the other hand, this uncertainty is unavoidable in a cross-sectional study, and we can be sure that diagnoses were generally correct, because these were based on the established Bárány criteria. Second, although boosted decision tree models in contrast to DNN have the advantage to indicate variable importance we found that decision trees had a slightly better accuracy, better precision but lower sensitivity than the DNN models, i.e., these models are better to predict absence of disease than presence. Arguably, this inferior result is due to the relatively low prevalence of MD and VM for this one-vs-all approach. Certainly, the multiclass approach, i.e., building models that can predict multiple classes of diagnoses at one time, is of superior clinical utility. Future approaches should strive for multicenter data collection to have a sufficient number of cases to address the problem. A multi-class approach for boosted decision trees seems to be an advisable solution for future models.

The correct diagnosis of spontaneous episodic vestibular syndromes is challenging in clinical practice. Modern machine learning methods might be the basis for developing systems that assist practitioners and clinicians in their daily treatment decisions.

## Electronic supplementary material

Below is the link to the electronic supplementary material.

Supplementary file1 (DOCX 158 kb)

## Data Availability

Data is available upon reasonable scientific request.
