# Correction to: Development and validation of a classification algorithm to diagnose and differentiate spontaneous episodic vertigo syndromes: results from the DizzyReg patient registry

**DOI:** 10.1007/s00415-020-10293-9

**Published:** 2020-11-26

**Authors:** Michael Groezinger, Doreen Huppert, Ralf Strobl, Eva Grill

**Affiliations:** 1 Institute for Medical Information Processing, Biometry and Epidemiology, Ludwig-Maximilians-Universität München (LMU), Marchioninistr. 15, 81377 München, Germany; 2 German Centre for Vertigo and Balance Disorders, University Hospital Munich, Campus Grosshadern, Munich, Germany; 3 Department of Neurology, Ludwig-Maximilians-University, Munich, Germany

## Correction to: Journal of Neurology 10.1007/s00415-020-10061-9

The original version of this article unfortunately contained a mistake. The given names and family names were interchanged.

The names should be:

Michael (given) Groezinger (family)

Doreen (given) Huppert (family)

Ralf (given) Strobl (family)

Eva (given) Grill (family)

The original article has been corrected.

