# Supplementary material for: Development and validation of a classification algorithm to diagnose and differentiate spontaneous episodic vertigo syndromes: results from the DizzyReg patient registry
Source: J Neurol. 2020 Jul 13;267(Suppl 1):160–7. doi: 10.1007/s00415-020-10061-9 (PMC7718195; doi:10.1007/s00415-020-10061-9)
Supplement: Supplementary file 1 — Supplementary file1 (DOCX 158 kb) [file 415_2020_10061_MOESM1_ESM.docx]

Development and validation of a classification algorithm to diagnose and differentiate spontaneous episodic vertigo syndromes: results from the DizzyReg patient registry

Groezinger, M. et al.

Supplementary material

Table 1: Summary description of variables used for training the classifiers for VM and MM, respectively.

| Summary panel | Examples | Total number of variables per panel |
| --- | --- | --- |
| Technical diagnostic variables | Results from audiometry | 6 |
| Sociodemographic variables | age, gender, education, | 5 |
| Items from Dizziness Handicap Inventory | “Because of your problem, are you afraid to leave your home without having someone accompany you?” | 2 |
| Patient history | Tinnitus, duration of vertigo attacks | 39 |
| Variables from neurological examination | Presence of headache | 6 |
| Variables from orthoptic examination | Examination of nystagmus, | 44 |
| Miscellaneous | noise as trigger | 3 |
| Total |  | 105 |


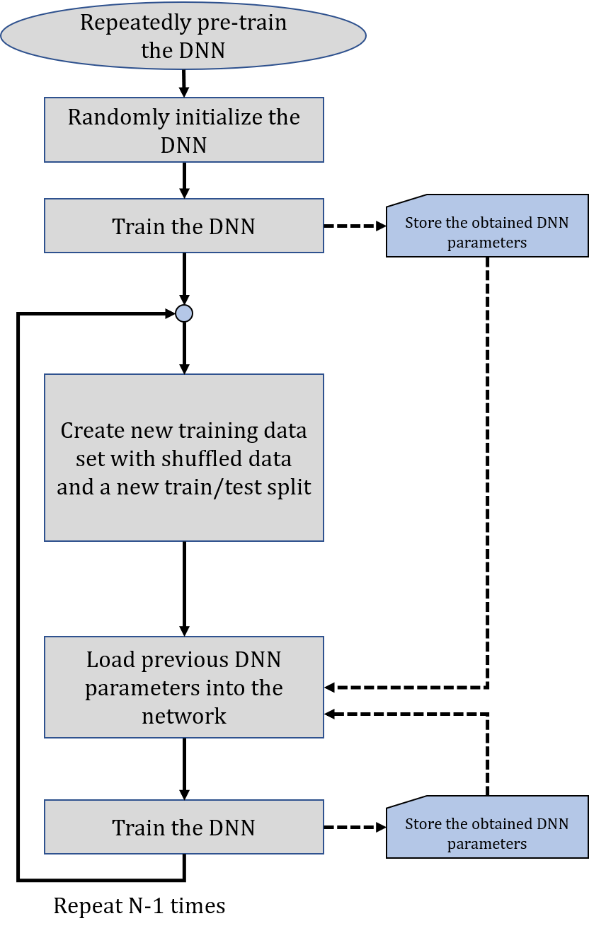


Figure 1: Description of the workflow for the repeated pre-training of Deep Neural Networks (DNN) to improve training accuracy
